# Supplementary material for: Role of Autoregulation and Relative Synthesis of Operon Partners in Alternative Sigma Factor Networks
Source: PLoS Comput Biol. 2016 Dec 15;12(12):e1005267. doi: 10.1371/journal.pcbi.1005267 (PMC5207722; doi:10.1371/journal.pcbi.1005267)
Supplement: S1 Text — (PDF) [file pcbi.1005267.s001.pdf]

S1 Text: Supplementary Methods and Derivations

Role of autoregulation and relative synthesis of operon partners in alternative sigma factor networks

Jatin Narula, Abhinav Tiwari and Oleg A. Igoshin

To understand the steady state post-translational response of σ^B network (Figs. 1, 2) we used the mass balance for the operon components RsbW, RsbV and σ^B together with the phosphate flux balance to derive approximate dependence of free σ^B on B_T. We found that the post-translational response of the network varies depending on whether the concentration of operon components is lower or higher than a threshold level defined by the concentration of the stress phosphatase P_T.

For low B_T ($[B_T] < 2[P_T]k_p/(k_k \min[\lambda_W, \lambda_V])$), the maximum phosphatase flux ($k_p[P_T]$) exceeds the maximum kinase flux ($k_k \min[[RsbW_T], [RsbV_T]]/2$) and as a result, $[V_P] \approx 0$. In addition most of the anti-anti-σ-factor V is in the W_2V_2 complex. Taking this into account and applying the mass balance for RsbV,

$$[RsbV_T] = [V] + [V_P] + [V_P P] + [W_2V] + 2[W_2V_2] \approx [V_P P] + 2[W_2V_2]$$

Next applying the balance for kinase and phosphatase fluxes,

$k_k\left([W_2V] + [W_2V_2]\right) = k_{deg}[V_P] + \left(k_p + k_{deg}\right)[V_P P]$ or, $k_k[W_2V_2] \approx \left(k_p + k_{deg}\right)[V_P P]$

Combining the above equations we find that $[W_2V_2] = \min\left[[RsbV_T]/\left(2 + k_k/\left(k_p + k_{deg}\right)\right), RsbW_T/2\right]$, where the minimum function is applied to account for the fact that the concentration of W_2V_2 cannot exceed half the total RsbW concentration.

Using the above equation in the mass balance for RsbW (while assuming that W, W_2 and W_2V are negligible) we can solve for W_2B and thereby σ^B,

$$2[W_2B] = RsbW_T - 2[W_2V_2] = [RsbW_T] - 2\min\left[[RsbV_T]/\left(2 + k_k/\left(k_p + k_{deg}\right)\right), [RsbW_T]/2\right]$$

$$[B_T] = [\sigma^B] + [W_2B] = [\sigma^B] + \max\left[[RsbW_T]/2 - [RsbV_T]/\left(2 + k_k/\left(k_p + k_{deg}\right)\right), 0\right]$$

$$
\begin{aligned}
[\sigma^{B}] &= [B_T] - \max\left[[RsbW_T]/2 \; - \; [RsbV_T]/\left(2 + k_k/\left(k_p + k_{deg}\right)\right), 0\right] \\
&= [B_T]\left(1 \; + \; \min\left[\lambda_V/\left(2 + k_k/\left(k_p + k_{deg}\right)\right) \; - \; \lambda_W/2, 0\right]\right) \\
&\approx [B_T]\left(1 \; + \; \min\left[\lambda_V/2 \; - \; \lambda_W/2, 0\right]\right)
\end{aligned}
$$

In contrast, for higher B_T ($[B_T] > 2[P_T]k_p/(\min\left(\lambda_W, \lambda_V\right)k_k)$), where the RsbW kinase dominates the phosphatase, V_P is not negligible and the phosphatase is saturated ($[V_P P] \approx [P_T]$). Again using this in the mass balance for RsbV,

$$
[RsbV_T] = [V] + [V_P] + [V_P P] + [W_2 V] + 2[W_2 V_2] \approx [V_P] + [V_P P] + 2[W_2 V_2]
$$

And applying the balance for kinase and phosphatase fluxes,

$$
\begin{aligned}
& k_k[W_2V_2] = k_{deg}[V_P] + \left(k_{deg} + k_p\right)[V_P P] \approx k_{deg}[V_P] + \left(k_{deg} + k_p\right)[P_T] \\
& \qquad [RsbV_T] = \left(k_k[W_2V_2] - \left(k_{deg} + k_p\right)P_T\right)/k_{deg} + [P_T] + 2[W_2V_2] \\
& [W_2V_2] = \left([RsbV_T] + [P_T]k_p/k_{deg}\right)/\left(2 + k_k/k_{deg}\right) \approx [RsbV_T]k_{deg}/k_k + [P_T]k_p/k_k \\
& 2[W_2B] = [RsbW_T] - 2[W_2V_2] = [RsbW_T] - 2[RsbV_T]k_{deg}/k_k - 2[P_T]k_p/k_k \\
& [B_T] = [\sigma^{B}] + [W_2B] \; = \; [\sigma^{B}] + [RsbW_T]/2 - [RsbV_T]k_{\mathrm{deg}}/k_k - [P_T]k_p/k_k \\
& [\sigma^{B}] = [B_T] + [RsbV_T]k_{\mathrm{deg}}/k_k + [P_T]k_p/k_k - [RsbW_T]/2 \\
& [\sigma^{B}] = [B_T]\left(1 + \lambda_V k_{\mathrm{deg}}/k_k - \lambda_W/2\right) + [P_T]k_p/k_k = [B_T]\left(1 + \lambda_V k_{\mathrm{deg}}/k_k - \lambda_W/2\right) + B_0\left([P_T]\right)
\end{aligned}
$$

where $B_0\left([P_T]\right) \; = \; [P_T]k_p/k_k$.

Note that since σ^B concentration cannot be negative this approximation only applies for $[B_T] < B_0\left([P_T]\right)/\left(\lambda_W/2 \; - \; 1 - \lambda_W k_{deg}/k_k\right)$. For higher B_T, $[\sigma^B]$~0.

Taken together the dependence of σ^B on B_T can be described by the following system of equations:

$$
[\sigma^B] = \begin{cases}
[B_T]\left(1 \; + \; \min\left[\dfrac{\lambda_V - \lambda_W}{2}, 0\right]\right) & \text{,if} \quad [B_T] < \dfrac{2[P_T]k_p}{\min[\lambda_W, \lambda_V]k_k} \\
[B_T]\left(1 + \dfrac{\lambda_V k_{deg}}{2k_k} - \dfrac{\lambda_W}{2}\right) + B_0\left([P_T]\right) & \text{,if} \quad \dfrac{2[P_T]k_p}{\min[\lambda_W, \lambda_V]k_k} < [B_T] < \dfrac{B_0\left([P_T]\right)}{\left(\lambda_W/2 \; - \; 1 - \lambda_W k_{deg}/k_k\right)} \\
-0 & \text{, if} \quad [B_T] > \dfrac{B_0\left([P_T]\right)}{\left(\lambda_W/2 \; - \; 1 - \lambda_W k_{deg}/k_k\right)}
\end{cases}
$$

Based on the above equation, the sensitivity of the σ^B post-translational response depends on (λ_W, λ_V), i.e. the stoichiometry of operon components:

$$\frac{\partial[\sigma^B]}{\partial[B_T]} = \begin{cases} \left(1 + \min\left[\frac{\lambda_V - \lambda_W}{2}, 0\right]\right) & \text{,if} \quad [B_T] < \frac{2[P_T]k_p}{\min[\lambda_W,\ \lambda_V]k_k} \\ \left(1 + \frac{\lambda_V k_{deg}}{2k_k} - \frac{\lambda_W}{2}\right) & \text{,if} \quad \frac{2[P_T]k_p}{\min[\lambda_W,\ \lambda_V]k_k} < [B_T] < \frac{B_0([P_T])}{\left(\lambda_W/2 - 1 - \lambda_W k_{deg}/k_k\right)} \\ -0 & \text{, if} \quad [B_T] > \frac{B_0([P_T])}{\left(\lambda_W/2 - 1 - \lambda_W k_{deg}/k_k\right)} \end{cases}$$

Which shows that the (λ_W, λ_V) parameter space can be divided into three regions based on qualitative differences in the post-translational response.

Region I ($\lambda_W < 2 + \lambda_V k_{\deg}/k_k$ **):** $\partial[\sigma^B]/\partial[B_T] > 0$ and free σ^B increases as a function of B_T irrespective of P_T.
Region II ($2 + 2\lambda_V k_{deg}/k_k < \lambda_W < 2 + \lambda_V$ **):** $\partial[\sigma^B]/\partial[B_T] > 0$ for $[B_T] < 2[P_T]k_p/(k_k \min[\lambda_W,\ \lambda_V])$ and $\partial[\sigma^B]/\partial[B_T] < 0$ for $2[P_T]k_p/(k_k \min[\lambda_W,\ \lambda_V]) \le [B_T] < B_0([P_T])/\left(\lambda_W/2 - 1 - \lambda_W k_{deg}/k_k\right)$. Thus free σ^B concentration is a non-monotonic function of B_T.
Region III ($\lambda_W > 2 + \lambda_V$): $\partial[\sigma^B]/\partial[B_T] < 0$ and free σ^B concentration decreases as a function of B_T irrespective of P_T.

Thus the asymptotic description shows how relative synthesis rate of σ^B operon partners by controls the sign of post-translational response sensitivity ($\partial[\sigma^B]/\partial[B_T]$). Specifically it shows that $\partial[\sigma^B]/\partial[B_T] < 0$ is only possible in Region II where $2 + 2\lambda_V k_{deg}/k_k < \lambda_W < 2 + \lambda_V$. This implies that the overall feedback in the σ^B network can only be negative in Region II, thereby explaining why pulsatile responses are only seen for combinations sampled from this region (Fig. 1C-E). Note also that the boundary equations for Region II closely approximate the boundaries of the operon stoichiometry space calculated by sampling (λ_W, λ_V) combinations (Fig. 2D).

The asymptotic description also explains (Fig. S5) the observation that a threshold level of phosphatase is essential for pulsing [13]. As shown above, for (λ_W, λ_V) combinations from Region II, the upper limit $[B_T] < B_0([P_T])/\left(\lambda_W/2 - 1 - \lambda_W k_{deg}/k_k\right)$, with $B_0([P_T]) = [P_T]k_p/k_k$, bounds the highest level of B_T for which $\partial[\sigma^B]/\partial[B_T] < 0$.

However $[B_T] \geq v_0 / k_{\deg}$ where v_0 and k_{deg} are the basal rate of transcription and protein degradation/dilution rate respectively.
Consequently, $\partial[\sigma^B]/\partial[B_T] < 0$ in Region II only can be achieved when:

$$P_T > v_0 k_k \left(\lambda_W / 2 - 1 - \lambda_V k_{\deg} / k_k\right) / (k_p k_{\deg})$$

This defines the threshold level of phosphatase essential for $\partial[\sigma^B]/\partial[B_T] < 0$ and for the σ^B network to operate in a negative feedback regime. As a result, the σ^B network only pulses for phosphatase levels above this threshold. Note that this threshold level is proportional to both the basal level of σ^B operon expression and the ratio of kinase to phosphatase rate constants and increases as a function of the RsbW synthesis ratio λ_W (Fig. S5). This indicates that it represents the basal level of kinase flux that the stress-regulated phosphatase flux must exceed to trigger a response.

Mathematical model of competition between σ^B and σ^A

Model equations

The following set of equations that describe network dynamics of this extended model:

$$\frac{d[B_T]}{dt} = v_0 + v_B - k_{\deg}[B_T]$$

$$\frac{d[RsbW_T]}{dt} = \lambda_W v_0 + \lambda_W v_B - k_{\deg}[RsbW_T]$$

$$\frac{d[RsbV_T]}{dt} = \lambda_V v_0 + \lambda_V v_B - k_{\deg}[RsbV_T]$$

$$\frac{d[\sigma^B]}{dt} = v_0 + v_B - k_{b3}[W_2][\sigma^B] + k_{d3}[W_2\sigma^B] + k_{b4}[W_2\sigma^B][V] - k_{d4}[W_2V][\sigma^B] - k_{\deg}[\sigma^B]$$
$$- k_{bb}[\sigma^B][RNApol] + k_{db}[RNApol\text{-}\sigma^B]$$

$$\frac{d[W_2]}{dt} = k_d[W]^2 + (k_{d1} + k_{k1})[W_2V] + k_{d3}[W_2\sigma^B] - (k_{b1}[V] + k_{b3}[\sigma^B] + k_{\deg})[W_2]$$

$$\frac{d[V_P]}{dt} = k_{k1}[W_2V] + k_{k2}[W_2V_2] + k_{d5}[V_PP] - (k_{b5}[P] + k_{\deg})[V_P]$$

$$\frac{d[W_2V]}{dt} = k_{b1}[W_2][V] + (k_{d2} + k_{k2})[W_2V_2] + k_{b4}[W_2\sigma^B][V]$$
$$- (k_{d1} + k_{k1} + k_{b2}[V] + k_{d4}[\sigma^B] + k_{\deg})[W_2V]$$

$$\frac{d[W_2V_2]}{dt} = k_{b2}[W_2V_2][V] - (k_{d2} + k_{k2} + k_{\deg})[W_2V_2]$$

$$\frac{d[V_PP]}{dt} = k_{b5}[V_P][P] - (k_{d5} + k_p + k_{\deg})[V_PP]$$

$$\frac{d[RNApol\text{-}\sigma^{B}]}{dt} = k_{bb}[\sigma^{B}][RNApol] - k_{db}[RNApol\text{-}\sigma^{B}] - k_{bpb}[p_B][RNApol\text{-}\sigma^{B}]$$
$$+ k_{dpb}[RNApol\text{-}\sigma^{B}\text{-}p_B] + v_B[RNApol\text{-}\sigma^{B}\text{-}p_B] - k_{\deg}[RNApol\text{-}\sigma^{B}\text{-}p_B]$$

$$\frac{d[RNApol\text{-}\sigma^{B}\text{-}p_B]}{dt} = k_{bpb}[p_B][RNApol\text{-}\sigma^{B}] - k_{dpb}[RNApol\text{-}\sigma^{B}\text{-}p_B] - k_{\deg}[RNApol\text{-}\sigma^{B}\text{-}p_B]$$

$$\frac{d[RNApol\text{-}\sigma^{A}]}{dt} = k_{ba}[\sigma^{A}][RNApol] - k_{da}[RNApol\text{-}\sigma^{A}] - k_{\deg}[RNApol\text{-}\sigma^{A}]$$

$$[W_2\sigma^{B}] = B_T - [\sigma^{B}] - [RNApol\text{-}\sigma^{B}] - [RNApol\text{-}\sigma^{B}\text{-}p_B]$$

$$[W] = [RsbW_T] - 2[W_2] - 2[W_2\sigma^{B}] - 2[W_2V] - 2[W_2V_2]$$

$$[V] = [RsbV_T] - [W_2V] - 2[W_2V_2] - [V_P] - [V_PP]$$

$$[P] = [P_T] - [V_PP]$$

$$[p_B] = [p_B]_T - [RNApol\text{-}\sigma^{B}\text{-}p_B]$$

$$[\sigma^{A}] = [\sigma^{A}]_T - [RNApol\text{-}\sigma^{A}]$$

$$[RNApol] = [RNApol_T] - [RNApol\text{-}\sigma^{B}] - [RNApol\text{-}\sigma^{B}\text{-}p_B] - [RNApol\text{-}\sigma^{A}]$$

Model equations for the model of competition between σ^{B}*,* σ^{W} *and* σ^{A}

To model the competition for RNA polymerase between σ^{B} the housekeeping σ-factor σ^{A} and the alkaline stress response σ-factor σ^{W} (Figs. 6 and S8), we simplified the model for the post-translational control of stress σ-factors while explicitly including reactions for the binding/unbinding of RNA polymerase, σ-factors and target promoters. This model included the following set of equations:

$$\frac{d[B_T]}{dt} = v_{B0} + v_B - k_{\deg}[B_T]$$

$$\frac{d[W_T]}{dt} = v_{W0} + v_W - k_{\deg}[W_T]$$

$$\frac{d[\sigma^{B}]_{\text{free}}}{dt} = v_{B0} + v_B - k_{\deg}\left(1 + \frac{([B_T]/K_B)^{nb}}{[P_B]^{mb}}\right)[\sigma^{B}]_{\text{free}}$$

$$\frac{d[\sigma^{W}]_{\text{free}}}{dt} = v_{W0} + v_W - k_{\deg}\left(1 + \frac{([W_T]/K_W)^{nw}}{[P_W]^{mw}}\right)[\sigma^{W}]_{\text{free}}$$

$$\frac{d[RNApol\text{-}\sigma^{B}]}{dt} = k_{bb}[\sigma^{B}][RNApol] - k_{db}[RNApol\text{-}\sigma^{B}] - k_{bpb}[p_B][RNApol\text{-}\sigma^{B}] + k_{dpb}[RNApol\text{-}\sigma^{B}\text{-}p_B]$$
$$+ v_B[RNApol\text{-}\sigma^{B}\text{-}p_B] - k_{\deg}[RNApol\text{-}\sigma^{B}\text{-}p_B]$$

$$\frac{d[RNApol\text{-}\sigma^{B}\text{-}p_B]}{dt} = k_{bpb}[p_B][RNApol\text{-}\sigma^{B}] - k_{dpb}[RNApol\text{-}\sigma^{B}\text{-}p_B] - k_{\deg}[RNApol\text{-}\sigma^{B}\text{-}p_B]$$

$$\frac{d[RNApol\text{-}\sigma^{\mathrm{W}}]}{dt} = k_{bw}[\sigma^{\mathrm{W}}][RNApol] - k_{dw}[RNApol\text{-}\sigma^{\mathrm{W}}] - k_{bpw}[p_W][RNApol\text{-}\sigma^{\mathrm{W}}]$$

$$+ k_{dpw}[RNApol\text{-}\sigma^{\mathrm{W}}\text{-}p_W] + v_W[RNApol\text{-}\sigma^{\mathrm{W}}\text{-}p_W] - k_{\deg}[RNApol\text{-}\sigma^{\mathrm{W}}\text{-}p_W]$$

$$\frac{d[RNApol\text{-}\sigma^{\mathrm{W}}\text{-}p_W]}{dt} = k_{bpw}[p_W][RNApol\text{-}\sigma^{\mathrm{W}}] - k_{dpw}[RNApol\text{-}\sigma^{\mathrm{W}}\text{-}p_W] - k_{\deg}[RNApol\text{-}\sigma^{\mathrm{W}}\text{-}p_W]$$

$$\frac{d[RNApol\text{-}\sigma^{\mathrm{A}}]}{dt} = k_{ba}[\sigma^{A}][RNApol] \text{-} k_{da}[RNApol\text{-}\sigma^{\mathrm{A}}] \text{-} k_{\deg}[RNApol\text{-}\sigma^{\mathrm{A}}]$$

$$[\sigma^{\mathrm{B}}] = [\sigma^{\mathrm{B}}]_{\mathrm{free}} - [RNApol\text{-}\sigma^{\mathrm{B}}] - [RNApol\text{-}\sigma^{\mathrm{B}}\text{-}p_B]$$

$$[\sigma^{\mathrm{W}}] = [\sigma^{\mathrm{W}}]_{\mathrm{free}} - [RNApol\text{-}\sigma^{\mathrm{W}}] - [RNApol\text{-}\sigma^{\mathrm{W}} \text{ - } p_W]$$

$$[p_B] = [p_B]_T - [RNApol\text{-}\sigma^{\mathrm{B}}\text{-}p_B]$$

$$[p_W] = [p_W]_T - [RNApol\text{-}\sigma^{\mathrm{W}}\text{-}p_W]$$

$$[\sigma^{\mathrm{A}}] = [\sigma^{\mathrm{A}}]_T - [RNApol\text{-}\sigma^{\mathrm{A}}]$$

$$[RNApol] = [RNApol_T] - [RNApol\text{-}\sigma^{\mathrm{B}}] - [RNApol\text{-}\sigma^{\mathrm{B}}\text{-}p_B]$$

$$- [RNApol \text{ - } \sigma^{\mathrm{W}}] - [RNApol\text{-}\sigma^{\mathrm{W}}\text{-}p_W] - [RNApol\text{-}\sigma^{\mathrm{A}}]$$
