# Supplementary figures and images for: Role of Autoregulation and Relative Synthesis of Operon Partners in Alternative Sigma Factor Networks
Source: PLoS Comput Biol. 2016 Dec 15;12(12):e1005267. doi: 10.1371/journal.pcbi.1005267 (PMC5207722; doi:10.1371/journal.pcbi.1005267)

— Post-translational  
— Transcriptional

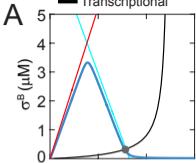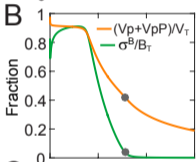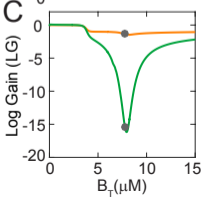

Supplement: S1 Fig — A. Decoupled post-translational (blue curve) and transcriptional (black curve) responses of the σB network for λW = RsbWT / BT = 4, λV = RsbVT / BT = 4.5. σB and BT represent the concentrations of free and total σB. Gray circle marks the steady states of the full system. Red and blue lines represent the piecewise analytical approximations of the post-translational response. B. Decrease in the fraction of phosphorylated RsbV (VP + VPP–orange curve) and unbound σB (green curve) as a function total operon expression level according to the post-translational response. C. Sensitivity of the post-translational response of phosphorylated RsbV (Vp+VpP—orange curve) and unbound σB (green curve) to changes in total operon expression level (BT). At the shown steady state (gray circles) both responses have LG<-1. (PDF) [file pcbi.1005267.s002.pdf]

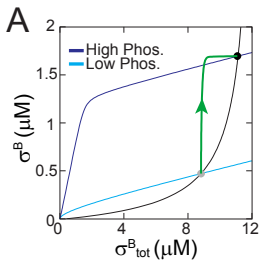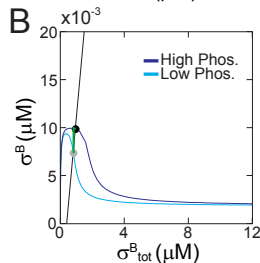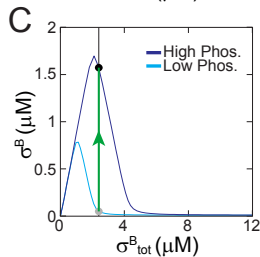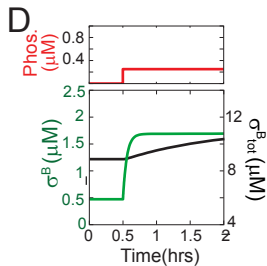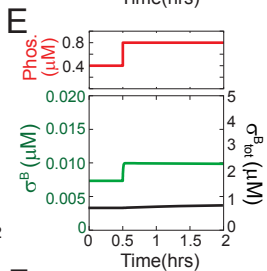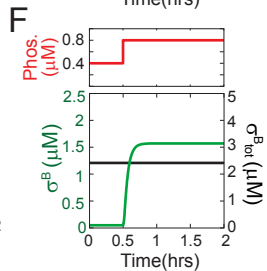

Supplement: S2 Fig — A-C. Decoupled post-translational and transcriptional responses of σB networks that lack negative feedback. (A) λW = 2, λV = 2 (Region I in Fig 2D)—positive feedback system (LG>0); (B) λW = 8, λV = 4.5 (Region III in Fig 2D) a non-responsive system (LG~0); (C) λW = 4, λV = 4.5 with no transcriptional feedback—no feedback system (LG = 0). In each panel cyan and blue curves show the post-translational response at low and high phosphatase concentrations, and black curve shows the transcriptional response. Gray and black circles mark the steady states of the full system. Step-increase in phosphatase causes a shift in the post-translational response from low phosphatase-cyan to high phosphatase-blue and leads to an increase in σB (green curve) in all three systems. D-F. Time-course representations of green trajectories described in A-C. Note that σB does not pulse in any of the three systems. (PDF) [file pcbi.1005267.s003.pdf]

**A**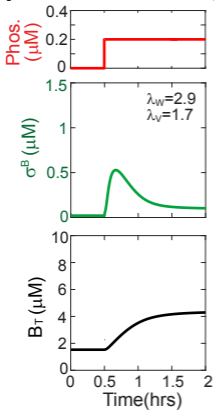**B**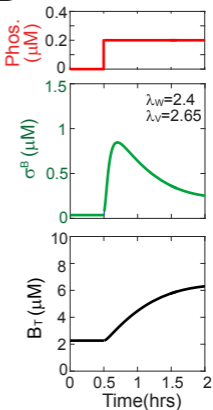

Supplement: S3 Fig — Dynamics of free σB in response to a step-increase in phosphatase concentration for the two different ratios measured in Delumeau et. al. [18]. (A) Pre-stress: λW = 2.9, λV = 1.7 (B) Post-stress: λW = 2.4, λV = 2.65. (PDF) [file pcbi.1005267.s004.pdf]

**A**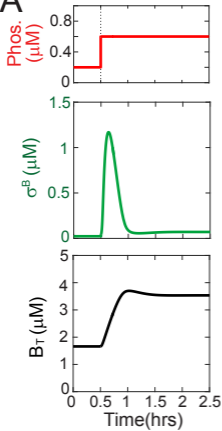**B**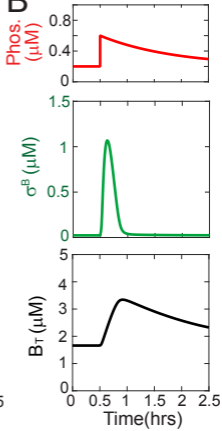

Supplement: S4 Fig — A. Dynamics of free σB in response to a step-increase in phosphatase concentration. B. Dynamics of free σB in response to a step-increase in phosphatase concentration which is followed by decay due to dilution. (PDF) [file pcbi.1005267.s005.pdf]

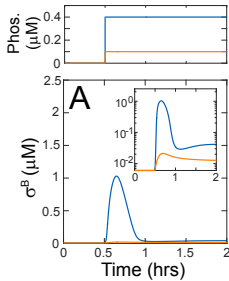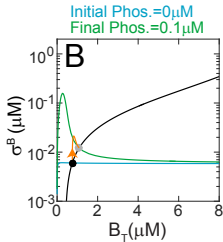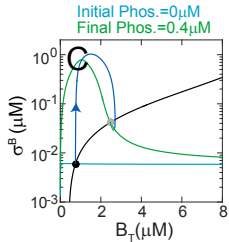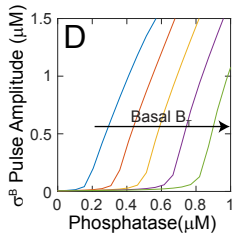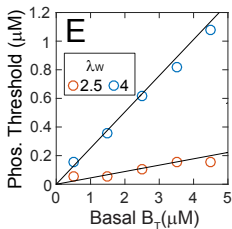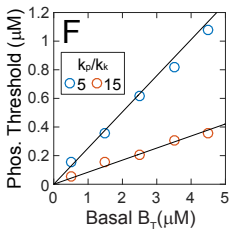

Supplement: S5 Fig — A. Time-course representations of σB pulse trajectories for small (0.1μM-orange curve) and large (0.4μM-blue curve) step-increases in phosphatase. λW = 4, λV = 4.5 for both trajectories. B,C. Representation of the σB pulse trajectories and decoupled post-translational and transcriptional responses of σB network for small (B) and large (C) step-increases in phosphatase. Cyan and green curves show the post-translational responses at initial and final phosphatase levels. Black curves show the transcriptional response. Black and gray circles mark the steady states of the full system. Note that at the initial phosphatase level the σB~0 and BT is at the basal level of σB operon transcription. The small step-increase in phosphatase does not significantly shift the post-translational response around the initial steady state leading to minor, transient increase in σB (orange curve in B). The large step-increase in phosphatase (C) does significantly shift the post-translational response around the initial steady state leading to prominent pulse in σB (blue curve in C). D. σB pulse amplitudes show a threshold linear response to increase in phosphatase level. The threshold phosphatase level increases with increasing basal level of σB operon transcription (Basal BT). E,F. Phosphatase threshold for pulsing as a function of Basal BT for different values of the (E) RsbW relative synthesis rate (λW) and (F) the ratio of phosphatase to kinase rates (kp / kk). The circles represent threshold levels calculated from simulations. The black lines represent the analytical approximation: PT = ν0kk (λW / 2–1 –λWkdeg / kk) / (kp / kdeg), where v0 is the basal rate of σB operon transcription and kdeg is protein degradation/dilution rate. Basal BT = ν0 / kdeg. (PDF) [file pcbi.1005267.s006.pdf]

**A**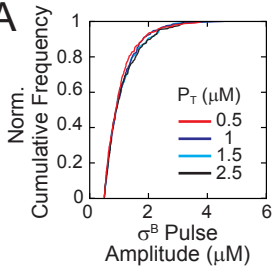**B**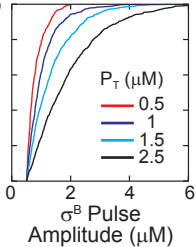**C**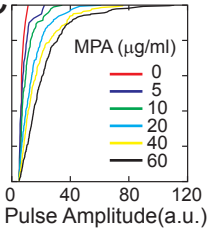

Supplement: S6 Fig — A-C. Normalized pulse amplitude cumulative histograms for stochastic simulations with (A) burst frequency modulation, (B) burst-size modulation and (C) experimental data taken from [13]. Different colors represent varying levels of mean phosphatase (PT) in the model or mycophenolic acid (MPA, energy stress) in experiments. (PDF) [file pcbi.1005267.s007.pdf]

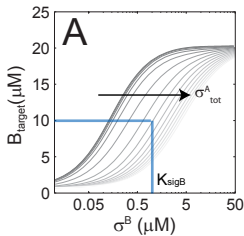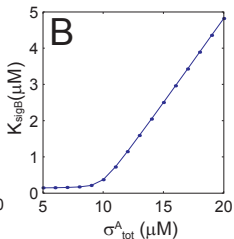

**C**

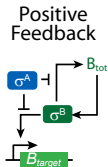

**D**

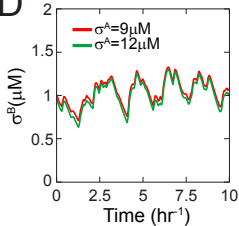

**E**

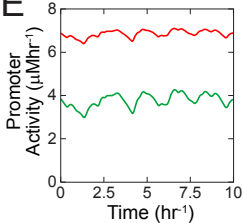

Supplement: S7 Fig — A. Steady-state dependence of σB target expression on free σB for different total levels of the housekeeping σ-factor (AT). B. KsigB, the half-maximal constant of the dependence of σB target expression, as a function of the total levels of the housekeeping σ-factor (AT). C. Simplified network diagrams of a positive feedback regulated stress σ-factor σB competing with housekeeping σ-factor σA for RNA polymerase. D-E. Trajectories of free σB (D) and σB target promoter activity (E) in response to stochastic phosphatase input at two different levels of total σA (AT = 9μM-low competition for RNA polymerase; AT = 12μM-high competition for RNA polymerase). (PDF) [file pcbi.1005267.s008.pdf]

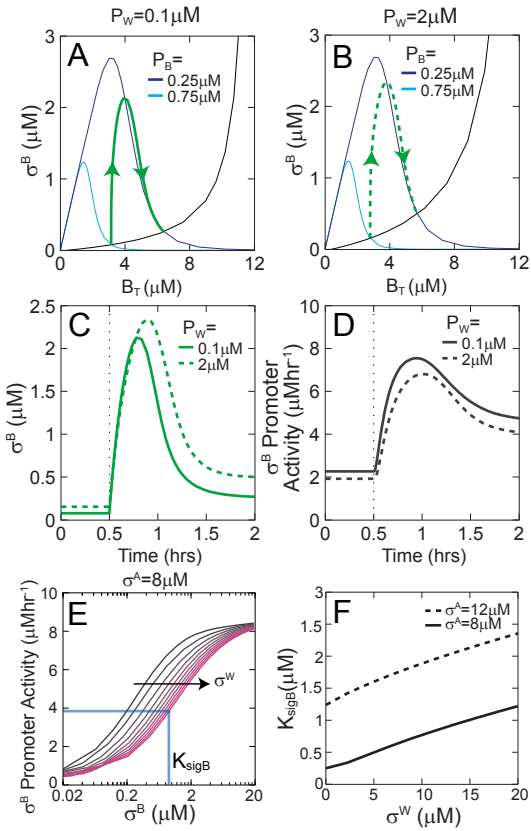

Supplement: S8 Fig — A,B. Decoupled σB post-translational and transcriptional components in the simplified model for the competition of stress σ-factors. Cyan and blue curves show the post-translational response at low and high concentration of σB stress signaling protein PB. Black curve shows the transcriptional responses. A step-increase in PB causes a shift in the steady-state post-translational response (from low phosphatase-cyan to high phosphatase-blue) and leads to a pulsatile σB response trajectory (green curve). Concentration of σW stress signaling protein PW was kept fixed at 0.1 μM (A) and 2 μM (B). C,D. Time-course representations of the green trajectories in (A,B) showing σB (C) and σB promoter activity (D) respectively. E. Steady state dependence of σB target promoter activity on the level of free σB for different levels of the σ-factor σW. F. KsigB, the half-maximal constant of the dependence of target expression on σB as a function of the concentration of the stress σ-factor σW for different levels of the housekeeping σ-factor σA. Total RNA polymerase core concentration was kept fixed at 10μM for all simulations. (PDF) [file pcbi.1005267.s009.pdf]
